# Apical Medium Flow Influences the Morphology and Physiology of Human Proximal Tubular Cells in a Microphysiological System

**DOI:** 10.3390/bioengineering9100516

**Published:** 2022-09-30

**Authors:** Gabriele Specioso, David Bovard, Filippo Zanetti, Fabio Maranzano, Céline Merg, Antonin Sandoz, Bjoern Titz, Federico Dalcanale, Julia Hoeng, Kasper Renggli, Laura Suter-Dick

**Affiliations:** 1 School of Life Sciences, University of Applied Sciences and Arts Northwestern Switzerland, 4132 Muttenz, Switzerland; 2 Philip Morris International (PMI) R&D, Philip Morris Products S.A., Quai Jeanrenaud 5, 2000 Neuchatel, Switzerland; 3 Swiss Centre for Applied Human Toxicology (SCAHT), 4056 Basel, Switzerland

**Keywords:** microfluidics, organ-on-chip, micro-physiological systems, cilia, kidney

## Abstract

There is a lack of physiologically relevant in vitro human kidney models for disease modelling and detecting drug-induced effects given the limited choice of cells and difficulty implementing quasi-physiological culture conditions. We investigated the influence of fluid shear stress on primary human renal proximal tubule epithelial cells (RPTECs) cultured in the micro-physiological Vitrofluid device. This system houses cells seeded on semipermeable membranes and can be connected to a regulable pump that enables controlled, unidirectional flow. After 7 days in culture, RPTECs maintained physiological characteristics such as barrier integrity, protein uptake ability, and expression of specific transporters (e.g., aquaporin-1). Exposure to constant apical side flow did not cause cytotoxicity, cell detachment, or intracellular reactive oxygen species accumulation. However, unidirectional flow profoundly affected cell morphology and led to primary cilia lengthening and alignment in the flow direction. The dynamic conditions also reduced cell proliferation, altered plasma membrane leakiness, increased cytokine secretion, and repressed histone deacetylase 6 and kidney injury molecule 1 expression. Cells under flow also remained susceptible to colistin-induced toxicity. Collectively, the results suggest that dynamic culture conditions in the Vitrofluid system promote a more differentiated phenotype in primary human RPTECs and represent an improved in vitro kidney model.

## 1. Introduction

The kidneys are complex organs that are vital in maintaining physiological body functions. The main role of the kidney is to regulate the fluid, electrolyte, and acid-base balances of the body to create a stable environment for tissue and cell metabolism. These life-sustaining functions are accomplished by glomerular filtration, resorption, and tubular secretion [1]. An equally important renal role is preserving nutrients while excreting metabolic waste products and xenobiotics. Since almost one-third of the most prescribed drugs undergo renal elimination [2], the organ is at particularly high risk for drug-induced kidney injury (DIKI) [3]. Although drugs can exert their toxic effects on various sites in the kidney through different cellular mechanisms, most xenobiotic secretion occurs in the proximal tubules, making them of considerable interest for nephrotoxicity studies [4]. Similarly, conditions such as polycystic kidney disease and other ciliopathies mainly affect tubular structures in the kidney [5].

The passage of substances through the kidney is an important factor in renal physiology, kidney disease, and DIKI. While drugs smaller than 50 to 65 kDa pass through the glomeruli in the unbound form, large or charged chemicals are often cleared from the circulation by transporter-mediated renal secretion in the tubules [4]. The main renal transporters belong to two superfamilies of carrier proteins: (1) ATP-binding cassette (ABC) proteins including P-glycoprotein (P-gp), breast cancer resistance protein (BCRP), and multidrug resistance-associated proteins (MRPs) and (2) solute carrier (SLC) proteins including organic anion transporters (OATs) and multidrug and toxin extrusion protein (MATEs) [6]. In addition, the recovery of proteins that pass glomerular filtration is mediated by megalin and cubilin. These receptors bind and retrieve low molecular-weight proteins such as albumin via clathrin-mediated endocytosis [7]. Aquaporin-1 (*AQP1*) is a water channel that is typically expressed in the proximal tubular cells of the kidney and is often used as a differentiation marker for renal proximal tubular epithelial cells (RPTECs) [8]. Appropriate function of these transporters is necessary for the kidney to perform its in vivo physiological function and is also a requirement for physiologically relevant in vitro kidney models.

Several in vitro systems have been established to perform preclinical studies and develop standardized assays to study the effects of drug-induced tubular injury using tissue slices, cell isolation methods, and tubular fragments, as well as cultures of established kidney cell lines [8,9]. Immortalized cell populations are commonly used but often fail to display many of the key phenotypic characteristics of primary RPTECs, such as the highly polarized columnar cell morphology observed in vivo [10]. Limited differentiation in established cell lines might partially be due to the lack of a physiological micro-environment that promotes kidney histodifferentiation. Indeed, the apical epithelial surface in the kidney proximal tubule is exposed to the shear stress generated by the constant flow of the glomerular filtrate in vivo. Systems able to generate perfusion cultures applied to the kidney cell cultures include the work by Minuth [11], Ferrell [12], and Brakeman [13]. Accordingly, Duan et al. demonstrated that fluid shear stress (FSS)-induced actin cytoskeletal reorganization and junctional formation in renal epithelial cells and altered expression of apical and basolateral transporters in cultured mouse proximal tubule cells [14,15]. Moreover, the presence of FSS modulates the remarkable characteristics of RPTECs and its physiological importance in the kidney has been shown by improved transporter expression [16]. FSS affects the cells via mechanosensors and this seems to involve the primary cilia. These organelles on the apical side of RPTECs act as a cellular sensor, provide information about the external environment, and mediate responses via several signaling pathways [17]. Defects in ciliary structure and/or function are associated with several human diseases and developmental disorders that are collectively called ciliopathies. Typical of the cilia is the acetylation of α-tubulin that does not occur in the cytoplasm. Deacetylation of α-tubulin by histone deacetylase 6 (HDAC6) has been implicated in ciliary disassembly in the context of various signaling events and in epithelial ciliary dysfunction in the lung as a consequence of cigarette smoke [18]. The control of the primary cilium is not yet fully elucidated; however, it is well known that cilia undergo morphological and functional adaptations in response to FSS [10]. In the kidney proximal tubule, FSS also modifies inter-cellular junctions, cell size, and cytokine secretion [19].

Micro-physiological systems and organ-on-chip approaches provide an opportunity for a paradigm shift in drug development [20,21]. They enable drug–drug interaction predictions, pharmacokinetic drug exposure studies, and potentially clinical trials on chip [22,23,24,25]. Despite the advances in this field, micro-physiological systems able to generate and maintain controlled flow conditions still need to be improved in terms of throughput. Numerous in vitro micro-physiological systems and organ-on-a-chip approaches have recently been established to culture RPTECs under conditions mimicking the physiological characteristics of the proximal tubule [26,27,28]. Despite major technological advances in culturing kidney cells (reviewed by Wilmer et al.), researchers have not yet established physiologically relevant kidney in vitro systems for disease modelling and toxicity assessment. Currently available cell lines are suboptimal models for RPTECs, and commonly used models lack many physiological stimuli, resulting in poor clinical prediction of DIKI.

**Figure 1:**
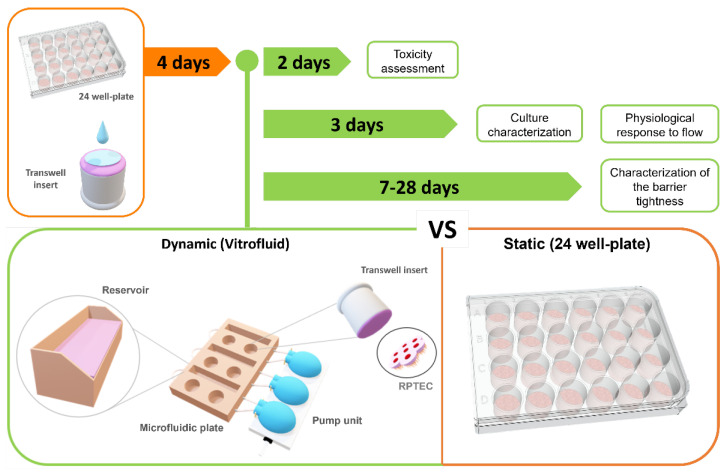
Overview and experimental timeline. RPTECs were cultured for 4 days under static conditions on the basal side of collagen-coated Transwell inserts (24 well-plates). Upon reaching confluence, some inserts were transferred to the Vitrofluid system to emulate a dynamic flow condition, and the remaining inserts were cultured in static conditions and used as controls. The responses to different doses of nephrotoxicants were evaluated on day two after administration. Characterization and the morphophysiological response of the cultures were studied on day three. Barrier tightness was characterized over 4 weeks.

Here, we established an in vitro model based on primary human RPTECs cultured within the dynamic Vitrofluid micro-physiological system (Figure 1). We employed primary cells because they resemble the phenotype of in vivo cells more closely than cell lines, in terms of morphology, polarization, drug transporter activity, and biomarker expression [29]. The Vitrofluid macro-format chip is made of a biocompatible and non-absorbent material, can house standard Transwell inserts, and be connected to a pump to generate a dynamic fluidic environment [30]. The model was characterized by determining morphological and functional parameters, as well as by assessing the susceptibility to colistin, which is a known inducer of DIKI [31].

## 2. Materials and Methods

### 2.1. Cell Culture

For the in vitro set-up of a human proximal tubule cell culture system, primary RPTECs were obtained from Merck (MTOX1030, Whitehouse Station, NJ, USA). RPTECs were used up to passage 6. A total of 3400 cells were seeded on the basal side of Transwell inserts (3470, Corning, Corning, NY, USA; diameter: 6.5 mm, 0.4-µm pore size) coated with collagen type I (08-115, Merck, Darmstadt, Germany) at a concentration of 70 µg/mL. RPTECs reached confluence after approximately 4 days. Cells were cultured in Eagle’s Minimum Essential Medium (M4526, Merck) supplemented with RPTEC complete supplement (MTOXRCSUP, Merck); L-glutamine solution (G7513, Merck), and gentamicin sulphate/amphotericin (CC-4083, Lonza, Basel, Switzerland).

**Figure 2:**
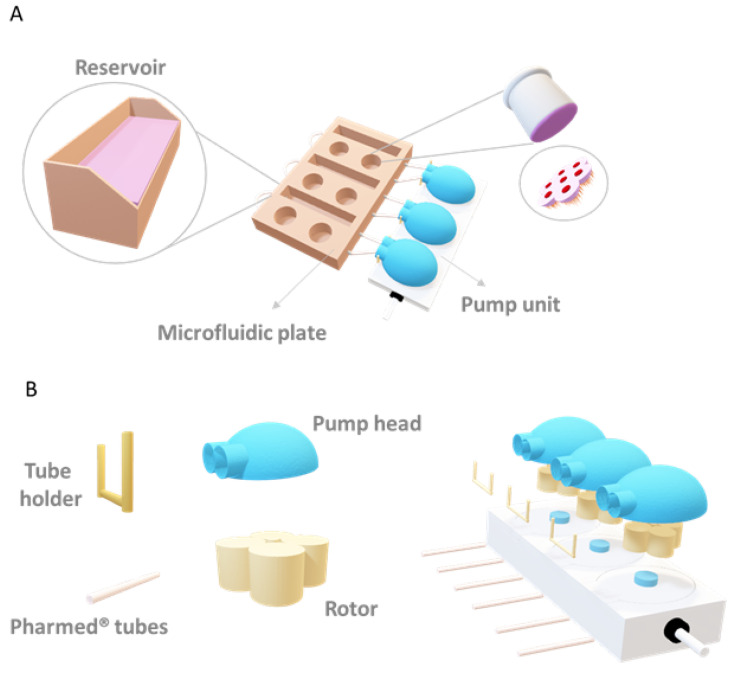
Schematic representation of the Vitrofluid system: (**A**) The Vitrofluid system consists of a pump unit with 3 pump heads connected to a microfluidic plate containing 3 reservoirs and 6 wells suitable for 24 well-plate Transwells. (**B**) Each pump head is composed of the cassette, rotor, PharMed tube, and tube holder. The flow used in these experiments (150 µL/min) led to an estimated FSS of 4.2 × 10^−4^ dyne/cm^2^, considered low flow conditions (see simulation Supplementary Figure S1).

Cells were kept for up to 28 days either under static conditions in Transwell inserts with medium exchange every 2 days or under flow by placing the inserts in the Vitrofluid system (Philip Morris Products S.A., Neuchâtel, Switzerland). The micro-physiological Vitrofluid system consists of three independent circuits, each with a pump and two wells to accommodate up to two Transwell inserts per circuit (diameter: 6.5 mm) and a medium reservoir (Figure 2). After preparing and connecting the microfluidic plate to the pump, 10 mL of culture medium was added to each reservoir, the pumps were set to a constant flow rate of 150 µL/min, and the Transwell inserts containing the cells were inserted in the wells of the microfluidic plate.

### 2.2. Cell Viability Assay

Cell viability was assessed with the Cell-Titer Glo^®^ Luminescent Cell Viability Assay 2.0 (G7570, Promega, Madison, WI, USA) according to the manufacturer’s instructions. Briefly, insert membranes were cut and treated with a 1:1 solution of distilled H_2_O and Cell-Titer Glo^®^. After 15 min at room temperature (RT) in a thermal mixer (400 rpm) (13687711, Thermo Fisher Scientific, Waltham, MA, USA), luminescence was measured on a plate reader (Flex Station 3, Bucher Biotec AG, Basel, Switzerland).

### 2.3. Membrane Permeance Assay

Lactate dehydrogenase activity was measured in the cell supernatant and cell lysates (MAK066, Merck) following manufacturer instructions. Briefly, 10 µL of cell culture medium or 5 µL of cell lysate were added to a solution of buffer and substrate provided in the kit in a clear bottom 96 well-plate (for a total of 100 µL/well). The enzymatic reaction was followed using kinetic measurement for 4 h at 450 nm at 37 °C. The data analysis was carried out using SoftMax Pro 5.4.5 software (Molecular Devices, San Jose, CA, USA).

### 2.4. Immunostaining

Cells on the insert were washed with phosphate-buffered saline (PBS, 11666789001, Merck) and fixed with 4% buffered formaldehyde (100496, Merck). Fixed cells were incubated overnight at 4 °C for 2 h at RT with the primary antibody diluted in PBS (containing 1% bovine serum albumin). Tested antigens were: acetylated tubulin (T7451, 1:4000, 6-11B-1, Merck), zona occludens 1 (ZO-1; 40300, 1:100, ZMD.437, Thermo Fisher Scientific), ezrin (PA5-82769, 1:100, Thermo Fisher Scientific), and Ki-67 (ab15580, 1:250, Abcam, Cambridge, UK). After washing, cells were incubated for 30–45 min at RT in the dark with the corresponding fluorescent labelled secondary antibodies: goat anti-rabbit IgG conjugated to Alexa Fluor 488 (A-11070, 1:1000, Thermo Fisher Scientific), goat anti-mouse IgG conjugated to Alexa Fluor 546 (A21143, 1:1000, Thermo Fisher Scientific), and goat anti-mouse IgG conjugated to Alexa Fluor 647 (A-21235, 1:1000, Thermo Fisher Scientific). Cell nuclei were subsequently stained with DAPI (0.1%, 2 min at RT).

For image acquisition, membranes were cut from the inserts, placed between two coverslips, and imaged using a fluorescence microscope (Axiovert 40 CFL, 036-42622, Zeiss, Oberkochen, Germany) or using a confocal laser scanning microscope (FluoView, FV3000, Olympus, Tokyo, Japan). The mean intensity and length were obtained using the software ImageJ (ImageJ 1.52a, National Institutes of Health, Bethesda, MD, USA).

### 2.5. Detection of Reactive Oxygen Species (ROS)

To detect ROS superoxide anions, MitoSOX™ Red Mitochondrial Superoxide Indicator was used as live-cell imaging staining (M36008, Thermo Fisher Scientific) following provider’s instructions. As a positive control, cultures were treated from the basal side for 5 min with 1% H_2_O_2_ (1.08600, Sigma-Aldrich, St. Louis, MO, USA) at 37 °C under 5% CO_2_).

### 2.6. Determination of Cilia Morphology and Orientation

RPTEC cilia morphology was observed under both static and dynamic conditions (Vitrofluid). Their length and orientation in relation to the flow direction were determined by immunostaining using an acetylated tubulin antibody (following the staining procedure described in Section 2.4) with ImageJ software assessment. To evaluate the effect of a change in medium flow direction, the Transwell inserts were maintained for 3 days in the Vitrofluid system under constant flow, then turned ~90° and maintained in dynamic culture conditions for 3 more days. The direction of the flow was marked directly on the polycarbonate membrane.

### 2.7. Albumin Uptake

Albumin uptake by RPTECs was determined on cells grown on collagen-coated Transwells under static and dynamic conditions. Cells were incubated with 200 µg/mL albumin-FITC (A9771, Merck) overnight and subsequently washed and fixed. To assess the specificity of the receptor-mediated uptake, some cultures were pretreated overnight with 200 µg/mL of the megalin/cubilin inhibitor cilastatin (SML1283, Merck).

### 2.8. Transferrin Uptake

After 7 days in culture under static and dynamic conditions, a solution containing 100 µg/mL of Alexa Fluor™ 546-conjugated transferrin (T23364, Thermo Fisher Scientific) was added to the basal compartment of RPTEC cultures. As a negative control, some RPTEC cultures were pre-incubated at 37 °C under 5% CO_2_ on an orbital shaker (120 rpm) for 15 min with a solution containing 1 mg/mL of unlabeled transferrin (T3309, Merck) to the basal compartment. After 1 h of incubation, cultures were washed 3 times for 5 min in PBS, and images were acquired using a fluorescence microscope.

### 2.9. Determination of Apparent Permeability

RPTEC barrier integrity was evaluated by determining the permeability to Lucifer Yellow after 7, 14, 21, and 28 days in culture. The apical medium was removed and replaced by 260 µL of 10 µg/mL Lucifer Yellow (LY; L0144, Merck). Immediately after this step, 130 µL of the apical solution was removed to determine the initial fluorescence (C_A0_), and the plates were placed on an orbital shaker set at 120 rpm for 1 h. Thereafter, the media from the apical and basal compartments were removed for fluorescence determination using a plate reader (Bucher Biotec Flex Station 3, Bucher Biotec, Basel, Switzerland) with settings for excitation at λex 428 nm and emission at λem 540 nm. The apparent permeability (P) was calculated using the equation: Papp (cm/s) = V_B_/(AC_AO_) × (ΔC_B_/ΔT), where V_B_ is the volume in the basolateral chamber, A is the surface area of the membrane, C_AO_ is the initial concentration in the apical chamber, and ΔC_B_/ΔT is the change of concentration in the basolateral chamber over time. Inserts without cells were used as baseline controls.

### 2.10. Gene Expression Analysis

Gene expression was determined using a quantitative reverse-transcriptase polymerase chain reaction (qRT-PCR) approach. Briefly, cells were lysed in TRIzol^®^ Reagent (15596026, Thermo Fisher Scientific) for RNA extraction. Total RNA from primary RPTECs was reverse transcribed at 37 °C for 1 h using M-MLV RT (M1705, Promega). cDNA was amplified using specific Taqman primers for the genes of interest and the housekeeping gene (Table 1). Relative gene expression was calculated as the ΔCT concerning the HK gene and expressed as 1/ΔCT. Total RNA directly extracted from human kidneys was used as a positive control (QS0616, Thermo Fisher Scientific) and prepared as described above.

### 2.11. Cytokine Detection

MILLIPLEX^®^ Human Cytokine/Chemokine/Growth Factor Panel A—Immunology Multiplex Assay (HCYTA-60K-12C, Merck KGaA, Darmstadt, Germany) was used for cytokine detection. Supernatants of RPTEC cultures kept under static or dynamic conditions for 7 days were used for detection of the following analytes: GRO (growth-regulated alpha protein, CXCL1), G-CSF (granulocyte colony-stimulating factor, CSF3), GM-CSF (granulocyte-macrophage colony-stimulating factor, CSF2), IL-1α (interleukin-1 alpha, IL1A), IL-1β (interleukin-1 beta, IL1B), IL-6 (interleukin-6, IL6), IL-8 (interleukin-8, IL8), IL-13 (interleukin-13, IL13), IFN-γ (interferon-gamma, IFNG), IP-10 (C-X-C motif chemokine 10, CXCL10), VEGF (vascular endothelial growth factor A, VEGFA), and TNF-α (tumor necrosis factor, TNF). The concentrations (in pg/mL) were normalized according to the number of cells and total volume.

### 2.12. Finite Element Simulation

The shear stress was simulated with Comsol Multiphysics v5.5. Due to the system symmetry only the simulation of a single well was necessary. The height of liquid in the well was set equal to 10 mm based on experimental measurements. The mass flow of 150 µL/min and the size of the channel allows to assume a laminar flow behavior of the fluid as the calculated Reynolds number is <10. As boundary conditions no-slip, laminar creeping flow, zero pressure at the outlet and fluid properties of water were used. The model was solved using physic controlled extra fine mesh. Shear stress was calculated from the velocity profile and liquid dynamic viscosity according to

$$\tau =\mu \times \frac{\partial v}{\partial z}$$

where *µ* is the dynamic viscosity and *δv*/*δz* is the velocity gradient or shear rate. As visible in Supplementary Figure S1, the RPTEC culture on the bottom of the transwell is subjected to a shear stress that can be estimated to be about 0.065 mPa or 6.5 × 10^−4^ dyne/cm^2^. The result is calculated for a 4.2 mm gap between transwell and the Vitrofluid well floor. The system allows simple variation of the shear stress not only by changing the flow rate but also by changing the dimensions of the transwell as a higher or lower gap significantly affects the velocity profile.

## 3. Results

### 3.1. Medium Flow Does Not Cause Cytotoxicity but Affects the RPTEC Plasma Membrane

**Figure 3:**
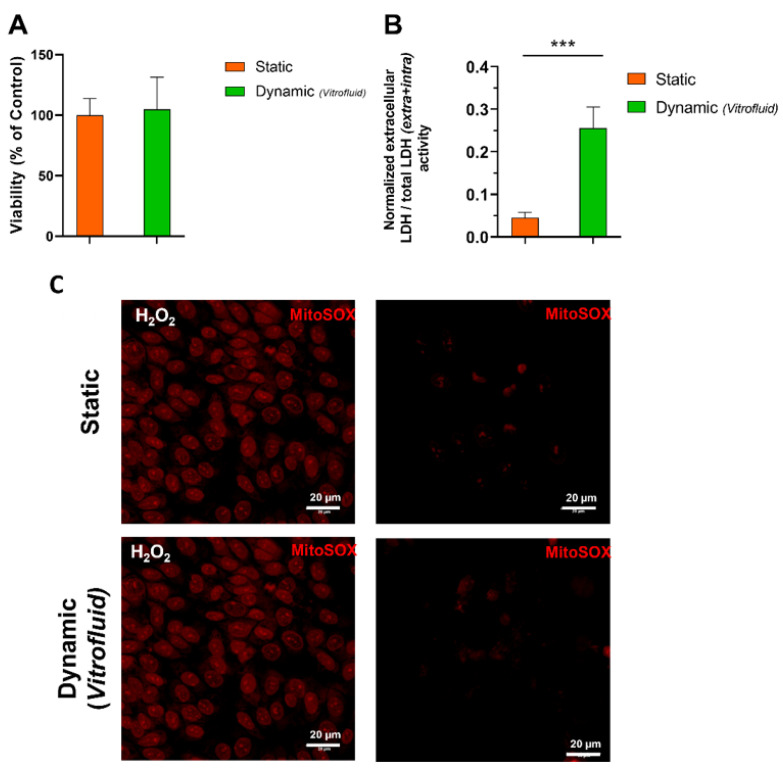
Viability of RPTEC cultures maintained for 7 days under static and dynamic conditions: (**A**) ATP content measured in RPTECs (mean ± SD; n = 3; Student’s *t*-test). (**B**) LDH concentrations in cell culture media (Mean ± SD; n = 3; Student’s *t*-test; *** *p* ≤ 0.001). (**C**) Superoxide detection with fluorescent staining with MitoSox Red^®^. Left panels: RPTECs were treated with 1% H_2_O_2_ as a positive control, right panels: untreated RPTECs (two biological repeats in technical triplicates).

The human kidney barrier model developed with human primary RPTECs under static (no flow) or dynamic (low flow) conditions remained viable for at least 1 week. After 7 days under unidirectional medium flow, cell viability determined by ATP content was unchanged (Figure 3A). In accordance with the lack of overt toxicity, cells exposed to flow for 7 days did not show any signs of oxidative stress, defined as the accumulation of superoxide in live cells and detected by staining with MitoSox Red (Figure 3C). In addition, expression of *HAVCR1* that encodes for the kidney injury molecule 1 (KIM-1), a biomarker of kidney toxicity, was not induced. The expression of this marker was significantly lower (0.47-fold) in RPTECs cultured under flow compared with cells cultured under static conditions (Supplementary Figure S2). Despite the lack of cytotoxicity observed, leakage of cytoplasmatic LDH into the medium was higher in RPTECs exposed to flow for 7 days compared to the static condition (Figure 3B). A significant increase in the ratio between extracellular and total LDH activity indicated an overall increased permeability of the plasma membrane of cells kept under flow.

### 3.2. Dynamic Culture Conditions Induce RPTEC Cytokine/Chemokine Secretion

**Figure 4:**
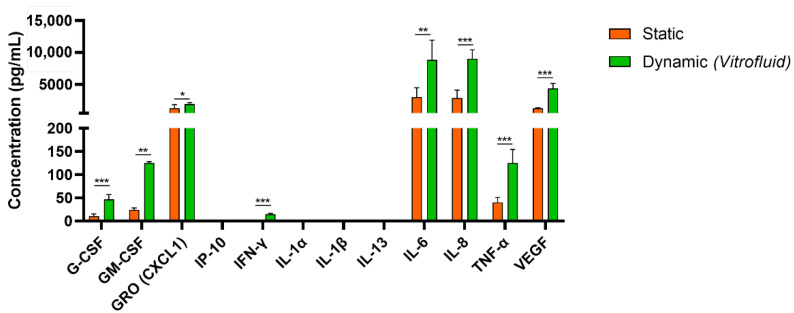
Cytokine secretion profile from RPTECs under static and dynamic conditions (day 7): Released cytokines were evaluated using a Luminex assay (mean ± SD; n = 2 biological repeats in technical triplicates; Student’s *t*-test; * *p* ≤ 0.05, ** *p* ≤ 0.01, *** *p* ≤ 0.001).

To further evaluate the effect of flow and the ensuing FSS on RPTECs, a panel of 12 cytokines/chemokines was quantified in RPTEC supernatant after 7 days in culture under dynamic or static conditions. In general, we observed that cultivation of the RPTECs under flow led to a significant increase in the secretion of the inflammatory cytokines TNF-α, IL-6, IL-8, and VEGF (approximately three times higher levels than in static conditions). Colony-stimulating factors (G-CSF and GM-CSF) were also significantly upregulated (4.4 and five times higher in dynamic than in static conditions, respectively). The immune cell-specific cytokines IP-10, IL-1a, IL-1β, and IL-13 were below the limits of detection (Figure 4).

### 3.3. RPTECs Maintain a Tight Barrier under Static and Dynamic Conditions

**Figure 5:**
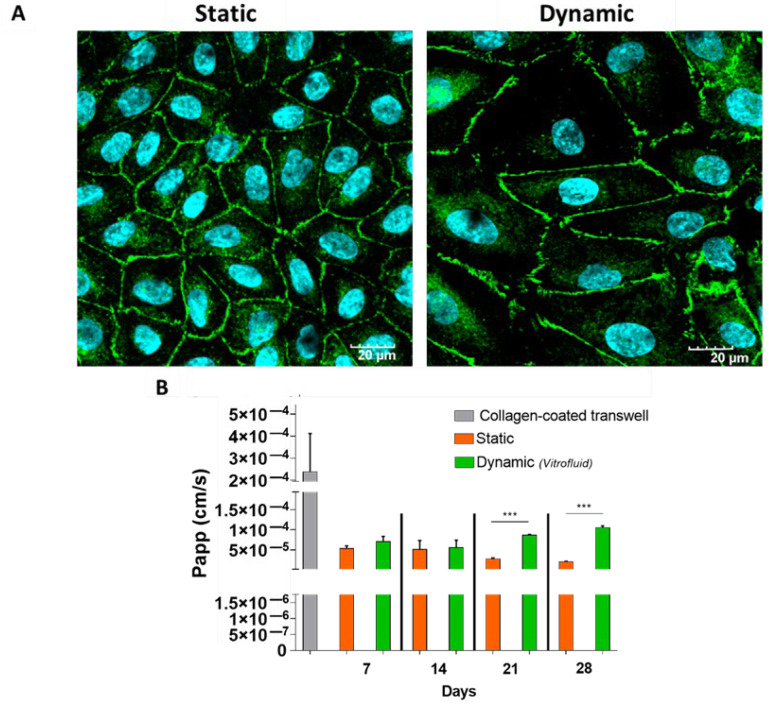
Time course characterization of barrier tightness under static and dynamic conditions: (**A**) Fluorescent staining of RPTECs to label tight junctions (ZO-1, green) and nuclei (DAPI, cyan) after 7 days in static or dynamic conditions. (**B**) Apparent permeability (Papp) measured after 1 h of incubation with 10 µg/mL Lucifer Yellow on days 7, 14, 21, and 28 (data are expressed as mean ± SD; n = 3; Student’s *t*-test; *** *p* ≤ 0.001).

The ability of the epithelial barrier of RPTECs to prevent passage of molecules from the apical to the basolateral side of the Transwells was determined by measuring the apparent permeability (Papp) to LY over time. After 7 days, the functionality of the cell layer under flow was comparable to that of RPTECs maintained in static conditions and the cells displayed a monolayer with well-developed tight junctions (Figure 5). Although our data show that barrier functionality could be partly maintained for up to 4 weeks under both static and dynamic conditions, FSS induced some barrier leakiness at 21 and 28 days (Figure 5).

### 3.4. Effect of FSS on Specific Transporters in RPTECs

**Figure 6:**
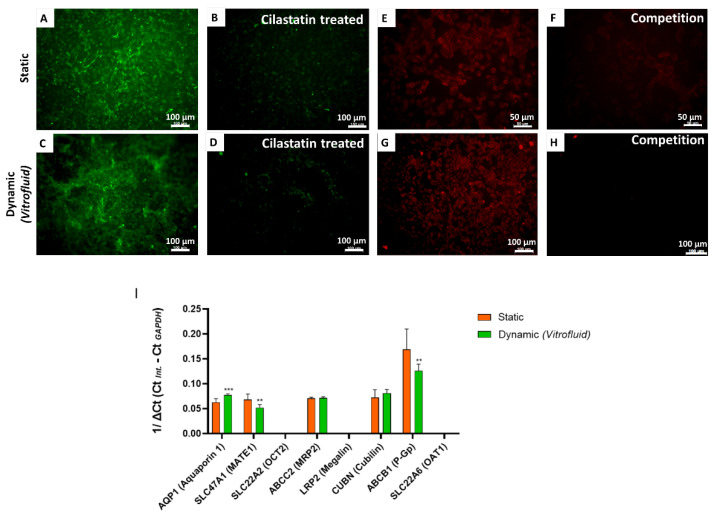
Albumin and transferrin uptake in RPTEC cultures under static and dynamic conditions on day 7: Cellular uptake of albumin-FITC (green) in the presence (**B**,**D**)/absence (**A**,**C**) of cilastatin (inhibitor of megalin/cubilin-mediated uptake). Cellular uptake of transferrin 546 (red) in the presence (**F**,**H**) and absence (**E**,**G**) of excess unlabeled transferrin (competition assay). Control cultures were pretreated overnight with cilastatin (200 µg/mL; **B**–**D**) or for 15 min in a solution of unlabeled transferrin (1 mg/mL; **F**,**G**). (**I**) Gene expression of renal transporters in RPTECs under static and dynamic culture conditions: Transporter expression was evaluated on day 7. Data are shown as 1/ΔCt. *GAPDH* was used as housekeeping gene (mean ± SD; n = 3 biological repeats in technical triplicates; Student’s *t*-test; ** *p* ≤ 0.01, *** *p* ≤ 0.001).

Once we confirmed that the model system cells built a tight barrier minimizing paracellular transport, we analyzed the expression of transporters that enable uptake and excretion of molecules for controlled transit across the RPTEC barrier. As shown in Figure 6A–H, RPTECs in both dynamic and static conditions could actively uptake albumin and transferrin. Notably, receptor-mediated uptake was reduced following co-incubation with cilastatin (for albumin), which is an inhibitor of the megalin/cubilin transporter, and by an excess of unlabeled transferrin, which competed with uptake of the labeled form.

In addition to functional uptake assays, we performed qRT-PCR to analyze the expression levels of several renal transporters. Gene expression data revealed elevated expression of aquaporin-1 (*AQP1*) in perfused cultures subjected to FSS compared with cells cultured under static conditions for 7 days. On the other hand, slightly decreased transcriptional levels of *SLC47A1* (MATE-1) and *ABCB1* (P-gp) were recorded under dynamic conditions. *SLC22A2* (OCT-2), *LRP2* (Megalin), and *SLC22A6* (OAT-1) expression were not detectable under any of the tested conditions (Figure 6I).

### 3.5. FSS Influences RPTEC Proliferation and Differentiation

**Figure 7:**
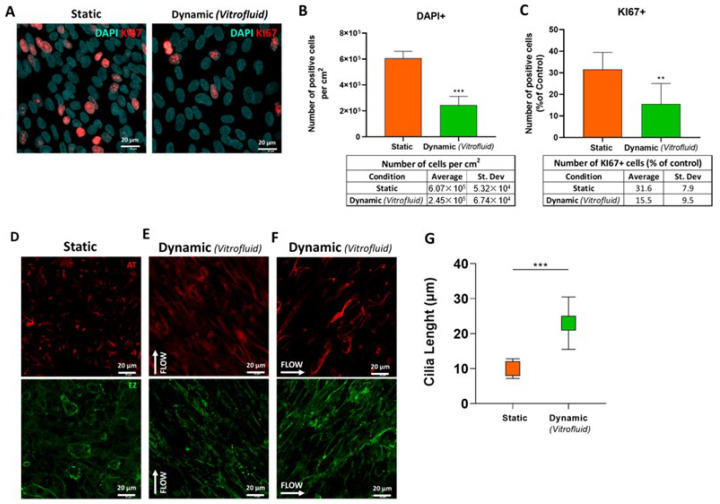
Effect of FSS on cell proliferation, cilia morphology and orientation: (**A**) Proliferating RPTECs under static and dynamic culture conditions on day 7 were stained with Ki67 (red) and DAPI (nuclei, cyan); n = 2 biological repeats in technical triplicates; Student’s *t*-test. (**B**) Average of RPTEC nuclei per cm^2^ cultured under static and dynamic conditions on day 7 (mean ± SD; three biological repeats in technical triplicates; Student’s *t*-test; *** *p* ≤ 0.001). (**C**) Average of Ki67+ nuclei after 7 days under static or dynamic conditions shown as a percentage of control (number of total nuclei per condition) (mean ± SD; two biological repeats in technical triplicates; Student’s *t*-test; ** *p* ≤ 0.01). (**D**–**F**) Immunostaining of acetylated tubulin (red) and ezrin (green) showing that cilia follow different flow directions: Static conditions (**D**), flow conditions for 3 days following the direction of the arrow €, and after rotation of the insert by 90° (as indicated by the arrow) and a further 3-day incubation (**F**). (**G**) Average cilia length measured with ImageJ software (mean ± SD; n = 3; Student’s *t*-test; *** *p* ≤ 0.001).

The increased expression of *AQP1* (Figure 6I) and decreased expression of *HAVCR1* encoding KIM-1 (Supplementary Figure S2) in cells exposed to unidirectional flow suggested enhanced RPTEC differentiation under flow. As differentiated cells generally proliferate less, we determined the proliferation rate of RPTECs in the presence and absence of FSS. Dynamic culture conditions for 7 days reduced RPTEC proliferation by two-fold in comparison with static conditions, as indicated by the number of Ki67-immunostained cell nuclei (Figure 7A,C). This was also reflected in the 2.5-fold reduction in the number of total cells per surface area (Figure 7B).

Culturing RPTECs under flow also promoted morphological changes in the cells, specifically cilia lengthening. As depicted in Figure 7D, medium flow led to marked changes in the morphology of the cilia, which were two times longer when grown under flow than in static conditions (23 ± 7 and 10 ± 2.8 µm, respectively) (Figure 7G). Cilia directionality was also influenced by the flow. After 3 days under dynamic conditions, all cilia aligned in the flow direction as depicted in Figure 7E. Upon rotating the cell culture inserts 90° within the Vitrofluid system, the cilia realigned with the new flow direction (Figure 7F).

Due to the observed morphological changes, we evaluated the effect of flow on the expression of *HDAC6*, as a potential modulator of cilia morphology. The quantitative analysis of gene expression levels showed significantly lower (0.51-fold) *HDAC6* expression in dynamic cultures compared to static ones (Supplementary Figure S2).

### 3.6. RPTECs under Flow Remain Susceptible to Colistin-Induced Toxicity

**Figure 8:**
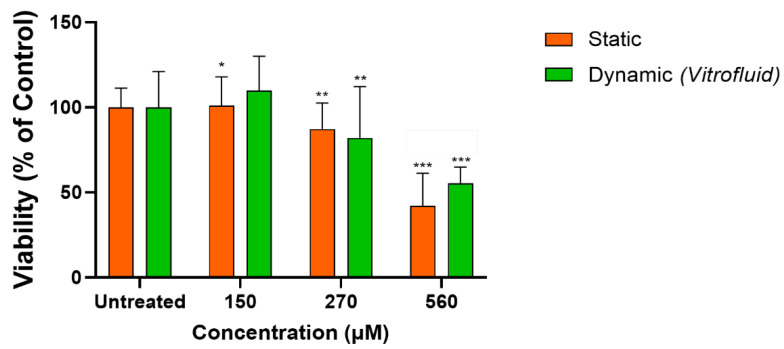
RPTEC culture response to different concentrations of the nephrotoxic antibiotic colistin under static and dynamic conditions: Viability of RPTEC cultures maintained for 4 days and subsequently exposed for 2 days to three concentrations of the nephrotoxic antibiotic colistin was measured by ATP assay. Data are shown as percentage of control and represented as mean ± SD and compared to the corresponding untreated cells (n = 2 biological repeats in triplicate technical repeats; * *p* ≤ 0.05; ** *p* ≤ 0.01; *** *p* ≤ 0.001).

RPTECs in conventional monolayer culture responded to the toxic compound colistin with the expected loss of cell viability and a half-maximal inhibitory concentration (IC_50_) of 562.6 µM determined by ATP assay (Supplementary Figure S3). Based on this result, we selected three different concentrations of colistin (150, 270, 562.6 µM) to compare the responses of RPTECs grown on membranes in dynamic or static conditions. At the highest tested concentration, the effect of colistin on RPTECs cultured under static conditions was very similar to that observed in conventional monolayer cultures (see Supplementary Figure S3), with approximately 50% loss in viability. At this concentration, cells cultured under dynamic conditions were significantly more viable (Figure 8).

## 4. Discussion

Several in vitro kidney models for drug screening have been described, but most have significant shortcomings [26]. This hinders the process of drug development, as investigation of the effects of disease and toxicity on renal function requires appropriate research systems. The kidney is a common target for toxicity as it is frequently affected by exposure to chemicals and drugs. Several studies have shown strong associations between the use of common drugs (e.g., antibiotics such as aminoglycosides and colistin) and clinically relevant nephrotoxicity [31]. Despite recent advances including organoid-based models, the lack of reliable human cell models limits preclinical research and the development of new drugs to treat primary ciliopathies [5]. Here, we report the establishment and characterization of an in vitro model of human proximal tubules, represented by human primary RPTECs cultured under dynamic conditions within the Vitrofluid system. Using this setup, we demonstrated that the functional characteristics of the epithelial barrier were maintained under constant flow and that FSS had profound effects on cellular morphology and behavior. We focused on the renal proximal tubule, as this structure is pivotal for transport functions and has been recognized as an important target of DIKI [32].

First, it was important to determine that the RPTEC cultures within the Vitrofluid system were viable under long-term FSS caused by exposure to constant, unidirectional flow. The data showed that human primary RPTECs were stable for assay purposes for 7 days. After 1 week, RPTECs cultured under flow did not show any signs of cellular stress as measured by intracellular ATP levels or intracellular ROS accumulation. The absence of ROS induction under the culture conditions is important since RPTECs are prone to oxidative stress due to the aerobic respiration required to support extensive active transport [33].

Our results indicated that RPTECs can be maintained in the Vitrofluid system under FSS for at least 1 week. In addition to the biochemical measurements, microscopic evaluation revealed a monolayer of cells that stained positive for ZO-1 and formed tight junctions that are typical of the proximal tubule [34]. This epithelial barrier was also functional, minimizing the passage of the fluorescent dye LY across the cell layer. Cells under both static and dynamic conditions maintained comparable barrier tightness, hindering the paracellular transport of LY for up to 14 days. After longer incubation periods (21 and 28 days) the barrier lost some of its tightness. For this reason, most subsequent experiments were performed in cells that were maintained under flow for 1 week.

The finding of increased extracellular LDH concentrations also suggests changes in the plasma membrane characteristics of RPTECs cultured in the Vitrofluid system. This leakage of LDH into the medium could not be explained by a loss of cell viability or increased cell numbers and is therefore likely due to changes in plasma membrane permeability. Others have demonstrated that plasma membrane structures such as tight junctions are not static structural elements but are dynamically regulated in diverse physiologic states [35]. Published evidence obtained from endothelial cells also indicates that the plasma membrane itself may act as a primary mechanotransducer, increasing its permeability [36] and microviscosity [37].

The RPTECs expressed many relevant transcripts encoding transporters required for the secretion of xenobiotics into the filtrate and the reabsorption of glucose and proteins such as albumin and transferrin. The transcription of *AQP1*, the principal water-transporting protein in the kidney proximal tubule, was significantly induced by the flow. This is particularly significant as *AQP1* expression in the proximal tubule is relevant for epithelial barrier establishment and potential responses to toxicants or drugs (e.g., hyperosmolarity agents such as Reno-60, Hypaque-76, and Visipaque) [38]. There is evidence for involvement of *AQP1* in proximal tubule cell migration and possibly in the structure’s response to injury [39]. The transcription of *ABCB1* (P-gp) was high in both the static and dynamic conditions, although the presence of flow significantly decreased its expression level. More controversial is the low expression of *SLC47A1* (MATE-1) under flow conditions, as this result contradicts data published by Fukuda et al., who described enhanced expression of this transporter in primary human RPTECs cultured under flow using a peristaltic pump system [40]. Similarly, maintaining RPTECs under dynamic conditions did not promote the expression of transporters such as *SLC22A2* (OCT-2), *SLC22A6* (OAT-1), and *LRP2* (megalin), which were not detectable even in the static condition. The lack of expression of specific transporters is a known limitation of in vitro kidney models and must be taken into consideration when assessing the effects of specific compounds. For example, the lack of functional OAT-1 may be an issue for the prediction of nephrotoxic effects of organic anions such as tenofovir or adefovir [41]. Similarly, a lack of expression of organic cation transporters (OCT-1) may decrease culture sensitivity to compounds like cisplatin [42]. In addition to the transport of anionic and cationic substances, proximal tubular cells are active in protein reabsorption. Our results demonstrate that RPTECs were able to perform receptor-mediated endocytosis of albumin and transferrin both in static and dynamic conditions. Albumin uptake could be blocked by the receptor inhibitor cilastatin [43], while the uptake of fluorescently labelled transferrin was decreased by incubation with excess unlabeled transferrin. Protein uptake was most likely mediated by the expression of cubilin, an essential protein involved in albumin reabsorption that could drive the uptake despite the absence of megalin [44].

Our extensive characterization of RPTECs cultured in the Vitrofluid system demonstrated that FSS does not cause cellular damage and may influence plasma membrane permeability, highlighting the importance of establishing quasi-physiological conditions in vitro. Moreover, RPTECs cultured under flow showed increased release of cytokines (IL-6, IL-8, TNF-α, G-CSF, and G-CSF) and growth factors (VEGF) into the medium. This is in agreement with recent reports that dynamic culture conditions increased RPTEC release of IL-6 and GM-CSF [45] and TNF-α and endothelial growth factor [19]. It is unclear which levels of cytokines are physiologically healthy or indicative of pathology; however, the ability to secrete cytokines indicates that flow-exposed RPTECs are physiologically responsive and could serve as a model to investigate renal inflammation, fibrosis, and nephrotoxicity.

Two key findings of this work are that apical exposure of RPTECs to unidirectional flow causes ciliary morphological changes and has an antiproliferative effect. In the kidney, the primary cilium length is dynamically altered by physiological factors including renal flow and the cell cycle, as well as pathological factors such as oxidative stress and cytokines [46]. Under physiologic conditions, cilia form when cells become quiescent and begin to disassemble prior to re-entering the cell cycle [47]. Thus, increased differentiation and cilia formation would be expected in RPTECs with a low proliferation rate, and our data demonstrate that FSS led to decreased cellular proliferation. Moreover, Park describes that cilia in differentiating cells are longer than in normal mature kidney tubular cells [46]. Our data show significantly longer cilia, concomitant with the transcriptional downregulation of *HDAC6* and a low proliferation rate in cells subjected to FSS compared to those cultured under static conditions. *HDAC6* has been identified as a driver of ciliary disassembly, and its activation is required in the context of various signaling events related to kidney disease [48] and is also involved in tracheal ciliary dysfunction in response to cigarette smoke [18,49]. Moreover, indirect evidence of a more differentiated state under dynamic conditions is the decreased expression of KIM-1. This protein is an injury marker for proximal tubular cells in vivo; it is rarely expressed in normal cells but is induced and shed upon acute kidney damage [50]. During the ensuing repair process, KIM-1 expression is upregulated as tubular cells become less differentiated and acquire an alternative phagocytic phenotype [51]. Taken together, the data indicate that flow conditions promote a more differentiated RPTEC phenotype, a lower proliferation rate, increased expression of *AQP1*, and decreased expression of *HDAC6* and KIM-1.

It is interesting to note that the cilia we observed under dynamic cultures were unusually long (approximately 23 µm), which may reflect a pathological status. In general, a primary cilium is typically 3 to 10 µm long in quiescent and fully differentiated cells [19,52] and a lengthening of the cilia has been associated with pathologies such as autosomal dominant polycystic kidney disease (ADPKD) [53]. The RPTECs in the Vitrofluid device exhibited functional cilia, as they were able to sense the direction of the flow: The cilia aligned in the direction of the flow after 3 days of exposure to apical FSS and realigned when the direction of flow changed by rotating the inserts with cells by 90°. Whether the model is a representation of a healthy or a more diseased state would require additional research. This is particularly true due to the low FSS generated within the system. The selection of the flow rate was based on empirical data on the performance of cells in culture. The values obtained by the modelling (see Supplementary Figure S1) are surprisingly low in comparison with the expected shear stress experienced in vivo, that has been reported to be around 1–2 dyne/cm^2^ [54]. More research would be needed to evaluate the effect of different FSS-intensities. Our data show that the RPTECs in vitro responded to the low flow within the system indicating that even low flow can have profound effects on cell phenotype.

A well-characterized, useful kidney in vitro model must be amenable for the detection of DIKI. Here, we could demonstrate that RPTECs culture in static and dynamic conditions was susceptible to colistin-induced DIKI. Colistin is an antibiotic known for its clinically relevant nephrotoxicity. The incidence of colistin-induced nephrotoxicity in the clinic can be as high as 60% when plasma concentrations exceed 2.5 mg/L (approximately 2 µM), but proximal tubular toxicity is probably due to an accumulation of the compound inside the cells, with potentially higher intracellular concentrations [55]. Exposure of the cells to colistin showed that RPTECs were sensitive to this known nephrotoxicant with an IC_50_ of 560 µM in conventional monolayer cultures. RPTECs cultured on Transwells under static and dynamic conditions showed responses at similar concentrations. Previous in vitro studies in proximal tubular cells reported toxicity of colistin with IC_50_ values in the high micromolar range (250 to 690 µM) [56,57], similar to the IC_50_ obtained in our system. The fact that the model can detect colistin-induced nephrotoxicity makes it an attractive alternative for research on substitute antibiotics, which is urgently needed for clinical use.

In this work, we demonstrated that exposure of primary RPTECs to constant flow in the Vitrofluid system for 1 week was not detrimental to the cells and induced important phenotypical changes. Some of the most striking effects of FSS were altered cell membrane permeability and changes in the length and orientation of the cilia. The results regarding the decreased proliferation rate, lower *HDAC6* and KIM-1 expression, and ciliary lengthening and alignment in the presence of flow strongly suggest that FSS promotes a more differentiated phenotype in RPTECs. Future refinement of the model could address the absence of expression of some transporters (OAT-1, OCT-2, and megalin) and investigating the impact of lengthened cilia. In conclusion, RPTECs cultured under flow were stable for at least 1 week and possessed key functions such as barrier tightness, albumin and transferrin uptake, and sensitivity to colistin-induced toxicity. Collectively, our results indicate that primary human RPTECs cultured in the Vitrofluid system are a suitable in vitro kidney model.

## Figures and Tables

**Table 1:** List of genes investigated by qRT-PCR, their encoded proteins, and the specific assay numbers from the provider (Thermo Fisher Scientific). *GAPDH* was used as the housekeeping gene.

Gene	Gene Name	Protein	Assay Number
Solute Carrier Family 22 Member 6	*SLC22A6*	Organic Anion Transporter 1 (OAT-1)	Hs00537914_m1
Solute Carrier Family 22 Member 2	*SLC22A2*	Organic Cation Transporter (OCT-2)	Hs00533907_m1
LDL Receptor Related Protein 2	*LRP2*	Megalin	Hs00189742_m1
ATP Binding Cassette Subfamily C Member 2	*ABCC2*	Multidrug Resistance- Associated Protein 2 (MRP2)	Hs00166123_m1
Aquaporin-1	*AQP1*	Aquaporin-1	Hs01028916_m1
Histone Deacetylase 6	*HDAC6*	Histone Deacetylase 6	Hs00997427_m1
Hepatitis A Virus Cellular Receptor 1	*HAVCR1*	Kidney Injury Molecule 1 (KIM-1)	Hs00273334_m1
Solute Carrier Family 47 Member 1	*SLC47A1*	Multidrug And Toxin Extrusion Protein 1 (MATE 1)	Hs00217320_m1
ATP Binding Cassette Subfamily B Member 1	*ABCB1*	P-Glycoprotein 1 (P-Gp)	Hs00184500_m1
Cubilin	*CUBN*	Cubilin	Hs00153607_m1
Glyceraldehyde-3-Phosphate Dehydrogenase	*GAPDH*	Glyceraldehyde-3-Phosphate Dehydrogenase	Hs02786624_g1

## Data Availability

Data is contained within the article or Supplementary Material.

## Supplementary Materials

The following supporting information can be downloaded at: https://www.mdpi.com/article/10.3390/bioengineering9100516/s1, Figure S1: Simulation of the shear stress on RPTECs cultured in the Vitrofluid system. Figure S2: Gene expression of *HDAC6* and *HAVCR1* in RPTECs under static and dynamic conditions; Figure S3: Response of RPTEC cultures to different concentrations of colistin under static conditions; Figure S4: Gene expression of renal transporters from RNA samples extracted from human kidney.
