# Supplementary material for: Apical Medium Flow Influences the Morphology and Physiology of Human Proximal Tubular Cells in a Microphysiological System
Source: Bioengineering (Basel). 2022 Sep 30;9(10):516. doi: 10.3390/bioengineering9100516 (PMC9598399; doi:10.3390/bioengineering9100516)
Supplement: Supplementary file 1 [file bioengineering-09-00516-s001.zip › bioengineering-1893311-supplementary.pdf]

Supplementary Information

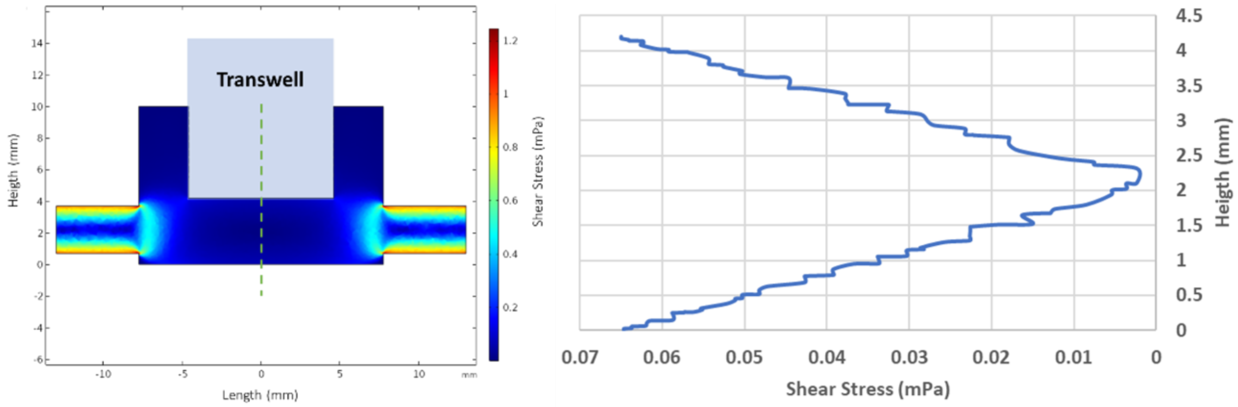

Figure S1. Simulation of the shear stress on RPTECs cultured in the Vitrofluid system: (left) shear stress heat map generated by a flow rate of 150 µL/min in the cross section of the Vitrofluid with a transwell placed 4.2 mm above the well floor. The color scale on the right is in mPa (range between 0 and 1.2 mPa). The dashed line indicates the central axis of the Vitrofluid well, where shear stress was calculated. (right) Shear stress calculated along the central axis with 4.2×10^{-4} dyne/cm^2 in average (range 4.6×10^{-5} and 6.5×10^{-4} dyne/cm^2).

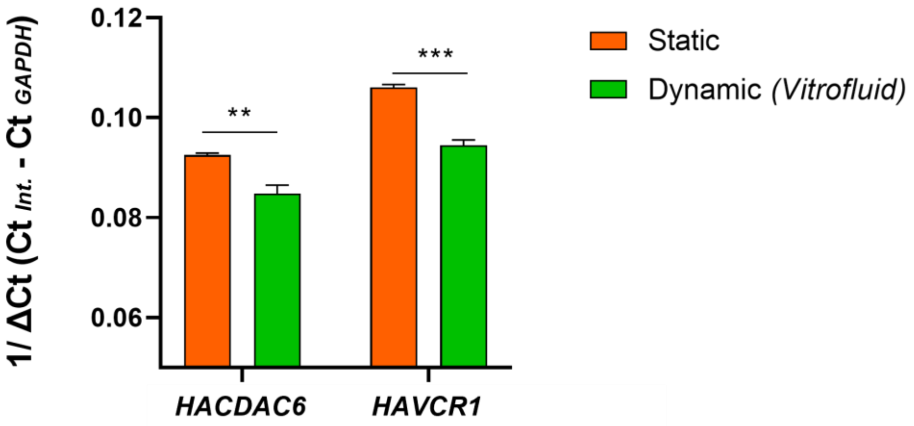

Figure S2. Gene expression of *HDAC6* and *HAVCR1* in RPTECs under static and dynamic conditions: *HAVCR1* and *HDAC6* expression were evaluated on day 7. Data are shown as 1/ΔCt. *GAPDH* was used as a housekeeping gene (mean ± SD; n = 2 biological repeats in technical triplicates).

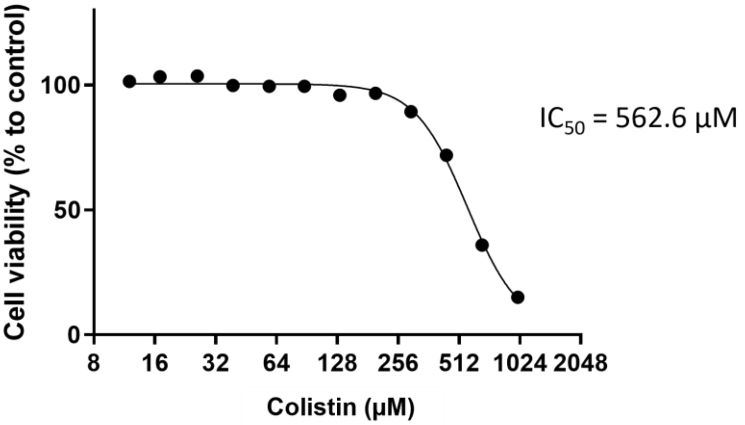

Figure S3. Response of RPTEC cultures to different concentrations of colistin under static conditions: IC_{50} value and dose-response curve of colistin measured by ATP assay in static RPTEC cultures exposed for 2 days. Data are shown as a percentage of control (untreated cells) and represented as mean ± SD (n = 2 biological repeats in triplicate technical repeats).

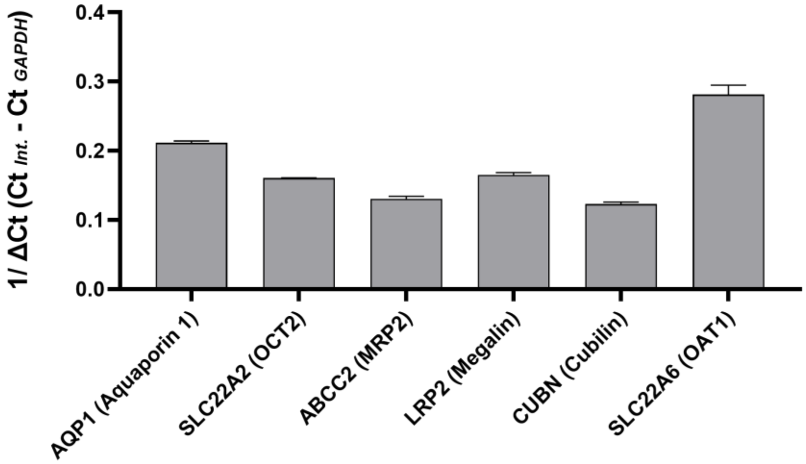

Figure S4. Gene expression of renal transporters from RNA samples extracted from human kidney: Total human kidney RNA (QS0616, Thermo Fisher Scientific) was used as a reference for expression levels of selected transporters. Data are shown as 1/ΔCt. *GAPDH* was used as a reference gene (data are represented as the mean ± SD of 3 technical triplicates).
